# Association between PSCA mRNA expression levels and rs2294008 polymorphism in transitional cell cancer of the bladder

**DOI:** 10.3892/ol.2014.2734

**Published:** 2014-11-24

**Authors:** KEWEN ZHENG, ZHIGANG CHEN, YE TIAN, GANGYUE HAO

**Affiliations:** 1 Department of Urology, Beijing Friendship Hospital, Capital Medical University, Beijing 100050, P.R. China; 2 Department of Urology, Peking Union Medical College Hospital, Chinese Academy of Medical Science and Peking Union Medical College, Beijing 100730, P.R. China

**Keywords:** bladder cancer, prostate stem cell antigen, rs2294008, single nucleotide polymorphisms

## Abstract

Prostate stem cell antigen (PSCA) was originally identified as a gene that is overexpressed in prostate cancer, and correlates with prostate cancer progression and prognosis. Recently, a significant association has been identified between the PSCA rs2294008 (C>T) polymorphism and the risk of developing bladder cancer. Therefore, the present study investigated the different expression levels of PSCA mRNA in transitional cell carcinoma (TCC) of the bladder and normal bladder tissue. Furthermore, the association between PSCA mRNA expression levels in TCC and different rs2294008 (C>T) genotypes and various clinicopathological features, including tumor stage and grade, were evaluated. Reverse transcription-quantitative polymerase chain reaction was performed on 80 TCC samples and 38 samples of normal bladder urothelium from TCC patients who underwent transurethral resection of bladder tumor or radical cystectomy at the Beijing Friendship Hospital (Beijing, China) between September 2010 and May 2011. Genomic DNA was extracted from tumor tissue and sequenced to determine the rs2294008 (C>T) genotype. PSCA mRNA expression was detected in all samples (100%); however, tumor samples exhibited significantly higher PSCA expression levels compared with the normal urothelium samples (P=0.038). PSCA mRNA expression was positively correlated with the histological grade of the tumor (G1-2 vs. G3; P=0.001); however, no significant difference was detected between patients with superficial (Ta or T1) and muscle-invasive (≥pT2) tumors (P=0.250). Thus, PSCA mRNA expression levels were associated with TCC and tumor histological grade, but not the tumor stage. Additionally, PSCA mRNA expression levels were significantly higher in T allele carriers compared with CC homozygous patients (P=0.001), indicating that the presence of the T allele may increase PSCA mRNA expression. Therefore, rs2294008 (C>T) may be associated with the biological properties of TCC and, thus, future research should focus on the physiological function of PSCA and the mechanism of rs2294008.

## Introduction

Transitional cell carcinoma (TCC) of the urinary bladder is the fourth most common solid tumor in males in the United States, with ~68,810 new cases reported annually in this region (1), and is the ninth most common solid cancer worldwide (2). Smoking tobacco and occupational exposure to chemical carcinogens have been established as the strongest risk factors for developing bladder cancer (3). Although numerous individuals are exposed to these risk factors, only a proportion develops bladder cancer, indicating that genetic susceptibility to bladder carcinogenesis may vary in the general population.

Previously, a genome-wide association study (GWAS) identified a significant association between the PSCA rs2294008 (C>T) polymorphism and the risk of bladder cancer in US and European populations (4). A subsequent study confirmed that this single-nucleotide polymorphism (SNP) was also associated with bladder cancer risk in the Chinese population (5). PSCA has been reported to be highly expressed in bladder cancer and, thus, has been considered as a useful marker in the diagnosis and progression of bladder cancer (6). Furthermore, the clinical use of PSCA in immunotherapeutic strategies has been evaluated (7,8).

PSCA is a cell surface antigen that belongs to the Ly-6/Thy-1 family of glycosylphosphatidylinositol-anchored cell surface antigens (9). Predominantly, PSCA is prostate-specific and expressed in a subset of basal and secretary cells of the healthy prostate. PSCA overexpression has been associated with an increase in tumor grade, stage, metastasis and recurrence in prostate cancer patients (10). The PSCA gene, located on chromosome 8q24.2, encodes a 123-amino acid protein with 30% homology to the immature lymphocyte cell surface marker SCA type 2. Furthermore, high PSCA gene expression has been identified in healthy extra-prostatic tissues, such as the bladder, esophagus, stomach, pancreas and kidney, as well as in other non-prostatic malignancies analogous to prostate cancer, including bladder, clear renal cell, gestational trophoblastic, pancreatic and non-small cell lung cancer (9).

PSCA protein expression has been reported in the transitional epithelium of the healthy bladder, and the majority of superficial and muscle-invasive TCC specimens exhibit high levels of PSCA expression (6,11). Furthermore, a previous study revealed that the T allele reduced the transcriptional activity of the PSCA promoter *in vitro* (12). Paradoxically, the T risk allele reduces PSCA transcription, whereas PSCA is overexpressed in bladder tumors. However, Fu *et al* (13) confirmed that the T risk allele of rs2294008 was associated with increased PSCA mRNA expression in normal and tumorous bladder tissue samples. On the basis of this, we conducted the present study.

Quantitative polymerase chain reaction (qPCR) is sensitive enough to detect low-level gene expression and accurate enough to quantify the full range of expression. The aim of the present study was to use this method to evaluate PSCA mRNA expression levels in TCC of the bladder and normal urothelium specimens, and to determine whether the rs2294008 polymorphism influences PSCA mRNA expression levels.

## Patients and methods

### Patients and tissue samples

The present study included 80 specimens of TCC of the urinary bladder and 38 specimens of normal urothelium from 80 patients who underwent surgery at the Beijing Friendship Hospital (Beijing, China) from September 2010 to May 2011. All patients were diagnosed with TCC of the urinary bladder. TCC tumor tissue was obtained from patients who underwent transurethral resection or radical cystectomy for bladder cancer, while normal urothelium samples were obtained from those who underwent radical cystectomy. Primary TCC tissue samples were obtained from tissue that grossly and clearly appeared to comprise a tumor of the urinary bladder. Tumor tissue samples from the same areas of tumor specimens and normal mucosa samples from patients undergoing radical cystectomy were stored in liquid nitrogen and confirmed by a senior pathologist of the Beijing Friendship Hospital. Tumor samples exhibiting marked inflammation or necrosis were excluded from further analysis. Tumors were staged according to International Union Against Cancer (14) and graded histologically according to the World Health Organization classification system (15). None of the patients had received previous intravenous chemotherapy or radiation. Patient and tumor characteristics, and the clinical outcome, are summarized in Table I. Informed consent for this study was obtained from all patients, and the study was approved by the Research Ethics Committee of the Capital Medical University.

### DNA extraction and genotyping

Genomic DNA was isolated from tumor tissue using a DNA extraction kit (QIAamp DNA mini kit; Qiagen, Valencia, CA, USA) according to the manufacturer’s instructions. PCR was performed to amplify the DNA, using PSCA rs2294008-specific primers, synthesized at the Biosune Biotechnology Co., Ltd. (Shanghai, China). The primer sequences were as follows: Sense, 5′-AAACCCGCTGGTGTTGACTGTG-3′ and anti-sense, 5′-CATCTCTGCCCATCCATCCGT-3′, producing a 459-bp product. The samples were amplified using the PCR Amplification kit (Takara Biotechnology Co., Ltd., Dalian, China), and each sample was prepared to a final volume of 20 μl, containing 1X PCR buffer, 4.0 mM MgCl_2_, 4.0 mM each deoxynucleotide, 0.4 μl each primer, 0.1 μl Takara Ex Taq polymerase (Takara Biotechnology Co., Ltd.) and 1–10 ng genomic DNA. PCR was performed under the following conditions: Denaturation at 94°C for 5 min; 35 cycles of denaturation at 95°C for 30s, annealing at 55°C for 30s and extension at 72°C for 30s; and a final extension at 72°C for 2 min in a Mastercycler^®^ ep gradient thermocycler (Eppendorf, Fujian, China). Amplicons of 459 bp were analyzed by electrophoresis in 3% agarose gel and visualized using the Molecular Imager^®^ Gel Doc™ XR+ system with Quantity One^®^ 1-D analysis software (Bio-Rad Laboratories, Hercules, CA, USA) following staining with ethidium bromide. The rs2294008 C>T polymorphism of PSCA was genotyped using sequencing by Biosune Biotechnology Co., Ltd.

### RNA isolation and RT reaction

Total cellular RNA was extracted from the tissue samples using an RNeasy Mini kit (Qiagen) according to the manufacturer’s instruction. Purified total RNA was assessed for purity by measuring the absorbance at 260 and 280 nm using the DU730 Nucleic Acid/Protein Analyzer (Beckman Coulter, Inc., Brea, CA, USA), and stored at −80°C prior to analysis. Total RNA was reverse-transcribed using a SuperScript^®^ III First Strand kit (Invitrogen, Carlsbad, CA, USA), according to the manufacturer’s instructions.

### qPCR for PSCA mRNA

To examine the mRNA expression levels of the target gene (PSCA) and the housekeeping gene (GAPDH), qPCR was performed using the Mastercycler ep realplex (Eppendorf) and SYBR^®^ Green I dye (Toyobo Co., Ltd., Osaka, Japan). The primer sequences were as follows: Sense, 5′-AAAGCCCAGGTGAGCAACGAG-3′ and anti-sense, 5′-CTGTGAGTCATCCACGCAGTTC-3 for PSCA, producing a 147-bp product; and sense, 5′-TCAAGATCATCAGCAATGCC-3′ and anti-sense 5′-TGTGGTCATGAGTCCTTCCA-3′ for GAPDH, producing a 100-bp product. Each PCR reaction (final volume, 10 μl) contained 1 μl complementary DNA, 0.6 μl primers and 4 mM MgCl_2_. The thermal cycling conditions for the PCR were as followed: Denaturation at 95°C for 5 min; and 40 cycles of denaturation at 95°C for 15 sec, annealing at 56.4°C for 20 sec and extension at 72°C for 20 sec. The PSCA and GAPDH sequences were amplified in duplicate from the tissue samples. To ensure that the correct product was amplified, all of the samples were separated by 3% agarose gel electrophoresis. The quantity of the target was calculated as the ratio of target gene to reference gene copies, to obtain a normalized value. A separate standard melting point curve for PSCA and GAPDH was constructed using a serial dilution gradient (range, 1/4^1^–1/4^5^) to determine the amplification efficiency. To verify that the melting curve results were correct, representative PCR products were sequenced.

### Statistical analysis

The PSCA rs2294008 allele frequency was calculated as: [1 × (h + 2H)]/2n, where ‘h’ represents the heterozygous genotype, ‘H’ the homozygous genotype and ‘n’ the sample size for each population. The differences between PSCA mRNA expression and various clinicopathological variables, such as stage and grade, were assessed using the Mann-Whitney U test. Values were presented as the median and the upper and lower quartiles (P75 and P25, respectively), and P<0.05 was considered to indicate a statistically significant difference. All statistical analyses were performed using SPSS software (version 16; SPSS, Inc., Chicago, IL, USA).

## Results

### Genotypes of rs2294008 and identification of the amplified product

**Figure 1:**
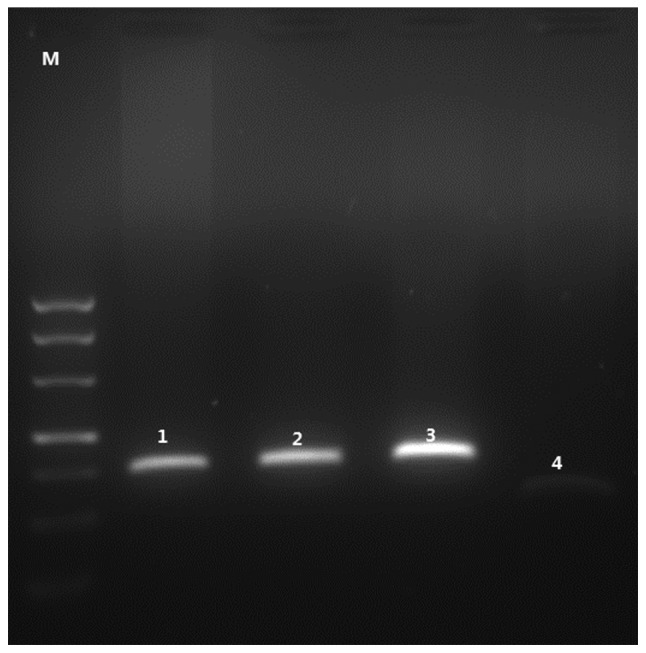
Agarose gel electrophoresis of the mRNA-polymerase chain reaction (PCR) products of prostate stem cell antigen and GAPDH. Lane M, DL500 DNA marker; lanes 1–3, mRNA-PCR products from PSCA (147 bp); lane 4: mRNA-PCR products from GAPDH (100 bp).

**Figure 2:**
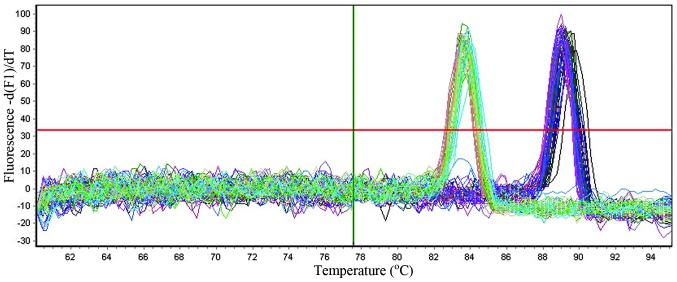
Melting curves for all of the polymerase chain reaction (PCR) products. The melting temperatures of GAPDH and prostate stem cell antigen (PSCA) were 83.5 and 89.0°C, respectively. PSCA and GAPDH are represented by one sharp peak each, indicating that only the specific PCR products were amplified.

The allele frequencies of rs2294008 C>T are demonstrated in Table II. Gel electrophoresis showed 147-bp PSCA and 100-bp GAPDH products (Fig. 1), and the melting curves for all of the PCR products only produced one sharp peak for PSCA and GAPDH, respectively (Fig. 2), indicating that the specific PCR products were amplified. Representative samples were sequenced to verify the correct products of PSCA and GAPDH.

### Amplification efficiency of PSCA and GAPDH

PSCA mRNA expression was detected in all samples (100%). The amplification products of PSCA and GAPDH were diluted in concentration gradients (range, 1/4^1^–1/4^5^) and amplified. The Ct values obtained were used to construct the standard melting curves. The Pfaffl method was used to calculate the amplification efficiencies of PSCA and GAPDH, which were 106.85 and 109.87%, respectively (16).

### Differences in PSCA mRNA expression

The PSCA mRNA expression levels in bladder cancer and normal urothelium tissues were normalized using GAPDH mRNA. RT-qPCR data was analyzed using the comparative Ct method. The following formula was applied: ΔCt = Ct_PSCA_ - Ct_GAPDH_, and the resulting 2^−ΔCt^ data exhibited a non-normal distribution. Therefore, the nonparametric, two independent samples, Mann-Whitney U test was performed.

Quantification of PSCA mRNA expression revealed a significantly greater expression of PSCA in TCC specimens compared with normal urothelium (P=0.038), and a significantly greater expression in G3 compared with G1-2 tumors of the bladder (P=0.001). No significant difference was identified in PSCA mRNA expression between patients exhibiting T1 and ≥pT2 tumors (P=0.250). Furthermore, T allele carriers demonstrated higher levels of PSCA mRNA expression compared with CC homozygous patients (P=0.001) (Table III).

## Discussion

Various studies have reported the potential importance of PSCA as a cell-surface antigen in the diagnosis and treatment of prostate, bladder, gastric and pancreatic cancers (6,17–22). Elsamman *et al* (6) reported that PSCA mRNA was expressed less in superficial TCC of the bladder in patients with disease recurrence compared with patients exhibiting no recurrence. Cheng *et al* (23) measured PSCA protein expression in urine samples, and demonstrated its possible application as a cytological marker of urothelial carcinoma via immunocytochemical analysis of urine. Subsequently, Cheng *et al* (11) used quantum dot-based technology to detect the levels of PSCA protein expression in human TCC, and revealed that PSCA expression correlated with tumor stage and grade. Furthermore, Kohaar *et al* (21) proposed that anti-PSCA immunotherapy may be beneficial for the treatment of bladder cancer patients with high tumor PSCA expression.

Numerous GWAS studies have been conducted to identify susceptibility loci of bladder cancer (4,24,25). One such study identified a significant association between the PSCA rs2294008 (C>T) polymorphism and the risk of bladder cancer in US and European populations (4). Ma *et al* (5) identified that this SNP was also associated with bladder cancer risk in the Chinese population. Notably, HapMap data indicates that the T allele is present at a higher frequency in individuals of European [minor allele frequency (MAF), 0.46] and Korean (MAF, 0.46) ancestry compared with Chinese (MAF, 0.26) and African (MAF, 0.25) populations (4). However, the T allele is a major allele in Japanese populations (MAF, 0.62). The population history of this SNP and the reason for only the Japanese population possessing a different minor allele is yet to be elucidated (4). rs2294008 is a missense SNP located in exon 1 of the PSCA gene (4). Linkage disequilibrium (LD) analysis of all HapMap SNPs in the vicinity of rs2294008 showed that it maps to an 11-kb LD block on chromosome 8q24 (4). Previously, an *in vitro* analysis of gastric cell lines determined that the T risk allele of rs2294008 was associated with a significant reduction in the transcriptional activity of the PSCA promoter (12). However, more recently, Fu *et al* (13) determined that the T risk allele was associated with increased PSCA mRNA expression levels in bladder tumor samples and normal bladder samples.

PSCA is overexpressed in bladder tumors. However, to date, few studies have been conducted to explain the association between the PSCA rs2294008 (C>T) polymorphism and mRNA expression in tumor tissue from TCC of the urinary bladder. The predominant aim of the present study was to evaluate the association between PSCA mRNA expression levels and rs2294008 (C>T) polymorphism and various clinicopathological features, including tumor stage and grade, and to determine whether the rs2294008 (C>T) polymorphism influences PSCA mRNA expression levels.

In the present study, PSCA mRNA was expressed at a significantly higher level in the tumor tissue of T allele carriers compared with CC homozygous patients (P=0.038). A previous study demonstrated that rs2294008 altered the length of the PSCA N-terminal signal peptide, which may affect protein folding, intracellular modifications and/or trafficking of PSCA proteins (4). However, the physiological function of PSCA, the impact of different N-terminal signal lengths on protein function and the *in vivo* functional consequence of the T risk allele remain unclear. Rs2294008 was considered to be an independent bladder cancer susceptibility locus on 8q24 (4). Additional studies are required to fully elucidate the physiological function of PSCA and the biological mechanisms underlying the association of the PSCA rs2294008 (C>T) polymorphism with bladder carcinogenesis.

In agreement with Amara *et al* (26), the present study identified a higher level of PSCA mRNA expression in TCC compared with normal urothelium. However, Bahrenberg *et al* (27) determined that PSCA expression levels were reduced in bladder cancer tumors and identified that PSCA expression was greater in confluent RT112 cells compared with non-confluent cells. In addition, Bahrenberg *et al* (27) reported that, in RT112 cells, PSCA expression is dependent on cell-cell contact and surface adhesion. In agreement with this, Elsamman *et al* (6) identified that confluent HT1376 TCC cells exhibited three-fold higher PSCA expression levels compared with the scattered (non-confluent) HT1376 cells. Therefore, the function of PSCA may be associated with the adhesion of cells, indicating that the expression level of PSCA correlates with tumor invasiveness. However, Amara *et al* (26) and Elsamman *et al* (6) reported that PSCA expression was inversely correlated with tumor stage. Instead, the greatest mean level of PSCA expression occurred in cases of superficial (Ta or T1) tumors, while lower PSCA expression levels occurred in cases of muscle-invasive (≥pT2) tumors. In the present study, no significant difference was identified between superficial (T1) and muscle-invasive (≥pT2) tumors (P=0.250), and patients with G3 tumors exhibited significantly higher PSCA mRNA expression levels compared with patients exhibiting G1-2 tumors (P=0.001). Research conducted by Kohaar *et al* (21) demonstrated a similar level of PSCA expression in non-muscle-invasive tumors, stages Ta and T1, and muscle-invasive tumors, stages T2 and T3/4. Furthermore, Elsamman *et al* (6) proposed that PSCA expression level is a predictor of disease recurrence in patients exhibiting superficial TCC of the urinary bladder, independent of tumor stage or grade.

In conclusion, the present study demonstrated that PSCA mRNA was more highly expressed in T allele carriers compared with CC homozygous patients, and PSCA mRNA expression was associated with TCC and tumor histological grade. Additional studies are required to define the physiological role of PSCA and the biological mechanisms underlying the association of the PSCA rs2294008 (C>T) polymorphism with bladder carcinogenesis.

## Figures and Tables

**Table I:** Patient and tumor characterics, and clinical outcome (mean patient age at surgery, 68.9 years; range, 48–92 years).

Variable	Patients, n (%) (n=80)
Gender	
Male	66 (82.5%)
Female	14 (17.5%)
Tumor stage	
pT1	54 (67.5%)
≥pT2	26 (32.5%)
Histological grade	
G1-2	56 (70.0%)
G3	24 (30.0%)
Surgical intervention	
Transurethral resection	42 (52.5%)
Radical cystectomy	38 (47.5%)

**Table II:** Genotypic and allelic frequencies for the prostate stem cell antigen rs2294008 allele T and C in transitional cell carcinoma of the 80 urinary bladder patients.

Variable	Patients, n (%)
Genotype	
CC	57.50
TC	32.50
TT	10.00
Allele	
C	73.75
T	26.25

**Table III:** Relative prostate stem cell antigen mRNA expression levels in various samples from 80 patients diagnosed with TCC of the urinary bladder.

Category	Samples, n	Median (P25, P75)	P-value
Tissue type			0.038
Healthy	38	0.12 (0.03, 0.54)	
TCC	80	0.22 (0.11, 0.84)	
Pathological grade			0.001
G1-2	56	0.25 (0.10, 0.47)	
G3	24	1.35 (0.69, 5.04)	
Pathological stage			0.250
T1	54	0.22 (0.10, 0.47)	
≥pT2	26	1.29 (0.36, 4.28)	
Genotype			0.001
CC homozygous	46	0.20 (0.10, 0.26)	
T carrier	34	0.48 (0.17, 1.59)	

TCC, transitional cell carcinoma; P25, lower quartile; P75, upper quartile.

## References

[1] Jemal A, Siegel R, Ward E (2008). Cancer statistics, 2008. CA Cancer J Clin.

[2] Jemal A, Murray T, Ward E (2005). Cancer statistics, 2005. CA Cancer J Clin.

[3] Corral R, Lewinger JP, Van Den Berg D (2014). Comprehensive analyses of DNA repair pathways, smoking, and bladder cancer risk in Los Angeles and Shanghai. Int J Cancer.

[4] Wu X, Ye Y, Kiemeney LA (2009). Genetic variation in the prostate stem cell antigen gene PSCA confers susceptibility to urinary bladder cancer. Nat Genet.

[5] Ma Z, Hu Q, Chen Z (2013). Systematic evaluation of bladder cancer risk-associated single-nucleotide polymorphisms in a Chinese population. Mol Carcinog.

[6] Elsamman E, Fukumori T, Kasai T (2006). Prostate stem cell antigen predicts tumour recurrence in superficial transitional cell carcinoma of the urinary bladder. BJU Int.

[7] Yang X, Guo Z, Liu Y (2014). Prostate stem cell antigen and cancer risk, mechanisms and therapeutic implications. Expert Rev Anticancer Ther.

[8] Marra E, Uva P, Viti V (2010). Growth delay of human bladder cancer cells by Prostate Stem Cell Antigen downregulation is associated with activation of immune signaling pathways. BMC Cancer.

[9] Saeki N, Gu J, Yoshida T, Wu X (2010). Prostate stem cell antigen: a Jekyll and Hyde molecule?. Clin Cancer Res.

[10] Lam JS, Yamashiro J, Shintaku IP (2005). Prostate stem cell antigen is overexpressed in prostate cancer metastases. Clin Cancer Res.

[11] Cheng F, Yu W, Zhang X, Ruan Y (2009). Quantum-dot-based technology for sensitive and stable detection of prostate stem cell antigen expression in human transitional cell carcinoma. Int J Biol Markers.

[12] Sakamoto H, Yoshimura K, Saeki N, Study Group of Millennium Genome Project for Cancer (2008). Genetic variation in PSCA is associated with susceptibility to diffuse-type gastric cancer. Nat Genet.

[13] Fu YP, Kohaar I, Rothman N (2012). Common genetic variants in the PSCA gene influence gene expression and bladder cancer risk. Proc Natl Acad Sci USA.

[14] Sobin LH, Gospodariwicz M, Wittekind C (2005). TNM Classification of Malignant Tumors. UICC International Union Against Cancer.

[15] Sauter G, Algaba F, Amin M, Eble JN, Sauter G, Epstein JI, Sesterhenn I (2004). Tumours of the urinary system: non-invasive urothelial neoplasias. WHO Classification of Tumours: Pathology and Genetics of Tumours of the Urinary System and Male Genital Organs.

[16] Chini V, Foka A, Dimitracopoulos G, Spiliopoulou I (2007). Absolute and relative real-time PCR in the quantification of tst gene expression among methicillin-resistant Staphylococcus aureus: evaluation by two mathematical models. Lett Appl Microbiol.

[17] Barbisan F, Mazzucchelli R, Santinelli A (2010). Expression of prostate stem cell antigen in high-grade prostatic intraepithelial neoplasia and prostate cancer. Histopathology.

[18] Yagn WB, Cai F, Cheng CT (2008). Expression of prostate stem cell antigen and Claudin-4 in human pancreatic carcinoma. Zhongguo Yi Xue Ke Xue Yuan Xue Bao.

[19] Antonarakis ES, Carducci MA, Eisenberger MA (2012). Phase I rapid dose-escalation study of AGS-1C4D4, a human anti-PSCA (prostate stem cell antigen) monoclonal antibody, in patients with castration-resistant prostate cancer: a PCCTC trial. Cancer Chemother Pharmacol.

[20] Krupa M, Canamero M, Gomez CE, Najera JL, Gil J, Esteban M (2011). Immunization with recombinant DNA and modified vaccinia virus Ankara (MVA) vectors delivering PSCA and STEAP1 antigens inhibits prostate cancer progression. Vaccine.

[21] Kohaar I, Porter-Gill P, Lenz P (2013). Genetic variant as a selection marker for anti-prostate stem cell antigen immunotherapy of bladder cancer. J Natl Cancer Inst.

[22] Sala N, Muñoz X, Travier N (2012). Prostate stem-cell antigen gene is associated with diffuse and intestinal gastric cancer in Caucasians: results from the EPIC-EURGAST study. Int J Cancer.

[23] Cheng L, Reiter RE, Jin Y (2003). Immunocytochemical analysis of prostate stem cell antigen as adjunct marker for detection of urothelial transitional cell carcinoma in voided urine specimens. J Urol.

[24] Kiemeney LA, Thorlacius S, Sulem P (2008). Sequence variant on 8q24 confers susceptibility to urinary bladder cancer. Nat Genet.

[25] Rothman N, Garcia-Closas M, Chatterjee N (2010). A multi-stage genome-wide association study of bladder cancer identifies multiple susceptibility loci. Nat Genet.

[26] Amara N, Palapattu GS, Schrage M (2001). Prostate stem cell antigen is overexpressed in human transitional cell carcinoma. Cancer Res.

[27] Bahrenberg G, Brauers A, Joost HG, Jakse G (2000). Reduced expression of PSCA, a member of the LY-6 family of cell surface antigens, in bladder, esophagus, and stomach tumors. Biochem Biophys Res Commun.

